# Screening Method for the Discovery of Potential Bioactive Cysteine-Containing Peptides Using 3D Mass Mapping

**DOI:** 10.1007/s13361-015-1282-z

**Published:** 2015-11-09

**Authors:** Luuk N. van Oosten, Mervin Pieterse, Martijn W. H. Pinkse, Peter D. E. M. Verhaert

**Affiliations:** Department of Biotechnology, Delft University of Technology, 2628 BC Delft, The Netherlands; Department of Biomedical Sciences, Antwerp University, 2610 Antwerp, Belgium; CEBMMS (Center of Excellence in Biological and Medical Mass Spectrometry), Department of Clinical Sciences, Lund University, 221 85 Lund, Sweden

**Keywords:** 3D mass mapping, Orbitrap analysis, Disulfide bridge containing peptides, Animal toxin, MATLAB script

## Abstract

Animal venoms and toxins are a valuable source of bioactive peptides with pharmacologic relevance as potential drug leads. A large subset of biologically active peptides discovered up till now contain disulfide bridges that enhance stability and activity. To discover new members of this class of peptides, we developed a workflow screening specifically for those peptides that contain inter- and intra-molecular disulfide bonds by means of three-dimensional (3D) mass mapping. Two intrinsic properties of the sulfur atom, (1) its relatively large negative mass defect, and (2) its isotopic composition, allow for differentiation between cysteine-containing peptides and peptides lacking sulfur. High sulfur content in a peptide decreases the normalized nominal mass defect (NMD) and increases the normalized isotopic shift (NIS). Hence in a 3D plot of mass, NIS, and NMD, peptides with sulfur appear in this plot with a distinct spatial localization compared with peptides that lack sulfur. In this study we investigated the skin secretion of two frog species; *Odorrana schmackeri* and *Bombina variegata*. Peptides from the crude skin secretions were separated by nanoflow LC, and of all eluting peptides high resolution zoom scans were acquired in order to accurately determine both monoisotopic mass and average mass. Both the NMD and the NIS were calculated from the experimental data using an in-house developed MATLAB script. Candidate peptides exhibiting a low NMD and high NIS values were selected for targeted de novo sequencing, and this resulted in the identification of several novel inter- and intra-molecular disulfide bond containing peptides.

**Electronic supplementary material:**

The online version of this article (doi:10.1007/s13361-015-1282-z) contains supplementary material, which is available to authorized users.

**Graphical Abstract:**
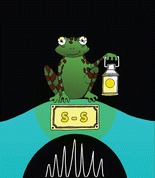
ᅟ

## Introduction

The venoms and toxins of animals are a major source of biologically active peptides and currently over 5000 of these peptides are recorded in the UniProt protein database [1, 2]. The nature of activity ranges from antimicrobial to neurotoxic, and this specific collection of peptides has proven to be an excellent source of potential new drug leads [3–5]. The classical route for the discovery of a bioactive peptide conventionally starts with determination of biological activity of the whole crude venom. Identification of individual peptides that are responsible for a certain activity is only achieved after extensive purification. Subsequent primary structure characterization either involves (de novo) sequencing via Edman degradation or tandem mass spectrometry (MS^n^). Cloning of messenger RNA encoding the bioactive peptides is another powerful approach [6], but only possible if some information of the primary sequence is known upfront.

Currently, the most widely used tool to study mixtures of non-tryptic endogenous peptides involves de novo sequencing from high resolution MS^n^ experiments using the ‘shotgun’ approach [7]. Characterization of animal toxin peptides via this approach is generally complicated because of the high number and high variety of post-translational modifications (PTMs), which makes de novo sequencing a difficult and time-consuming task [8, 9]. Fragmentation spectra can be ‘manually’ interpreted or analyzed using specialized algorithms for automated de novo sequencing such as PEAKS (Bioinformatics Solutions Inc., Waterloo, ON, Canada), PepNovo [10], and pNovo+ [11]. Full structure elucidation is only possible when complete fragmentation occurs and the spectrum is of sufficient quality with respect to signal-to-noise ratio. Instrumental parameters often need to be optimized for every peptide individually to yield spectra that meet these standards. Owing to the fact that a toxin sample can contain dozens of different peptides with accompanying truncated or partially degraded products, manually acquiring optimized tandem MS spectra for each individual peptide species and interpreting all their fragmentation spectra is a tremendously labor-intensive task. It is desirable to have several selection criteria to specifically target those peptides that meet these criteria upfront, before engaging in the time consuming task of de novo sequencing.

Remarkably, many of the biologically active peptides contain one or multiple disulfide bridges, which are believed to contribute to the peptides’ stability and activity. This is illustrated by the high prevalence of cysteines (6.91% of all residues) in the reviewed database from the animal toxin annotation program at UniProt compared with the whole UniProtKB/Swiss-Prot (release 2015_1) database (1.37% of all residues). The presence of disulfide bonds could thus be used as a selection criterion to target for unknown peptides in animal toxins and venoms with bioactivity potential.

A conventional way of determining the presence of disulfide bonds in a peptide is by simply counting the mass shift before and after reduction [12] or after reduction and alkylation [13]. Disadvantages of this approach are that it requires comparison of two separate analyses and that it is difficult to detect dimeric peptides in complex mixtures. Prior to sequence analysis of peptides, disulfide bonds are often reduced to improve the quality of MS^2^ spectra [14]. Additionally, free thiol groups of the cysteines can be derivatized using specific reagents such as iodoacetamide or selenamide [15, 16]. The main advantages of this type of sample preparation are that disulfide bridges cannot randomly reform and fragmentation of the modified peptide could yield higher sequence coverage [15, 16]. An additional advantage is that the derivatizing group could be engineered in such a way that cysteine-containing peptides can be specifically detected using tandem mass tags (TMT) [17, 18] in MS or even purified using isotope-coded affinity tags (iCAT) [19]. A disadvantage is that these labels are often relatively large and will negatively influence fragmentation, making de novo sequencing even more difficult. Specific isolation, such as in the case of iCAT, requires substantial amounts of starting material, which is not easy to obtain in the case of animal venoms. Another disadvantage with these labeling strategies is that it requires reduction of the disulfide bonds and, thereby, secondary structure information of dimeric peptides is lost.

In order to quickly assign the presence of cysteines in a peptide of unknown nature without the need of reduction of disulfide bond, we propose here to use a high resolution mass filtering approach. Two intrinsic characteristics of the sulfur atom can be used to select peptides with cysteine residues. Sulfur has a relatively large negative mass defect (the difference between the isotopic and nominal or integer mass) and it has a positive isotopic shift (difference between average and monoisotopic mass) [20]. Normalization of these two shifts results in two non-additive and independent peptide properties, which were previously introduced as normalized nominal mass defect (NMD) and normalized isotopic shift (NIS) by Artemenko et al. [21]. Certain types of peptides are localized in distinct regions on a 2D plot of NMD versus NIS, due to different chemical composition. For example, NMD tends to increase due to very basic (arginine, lysine) and aliphatic (leucine, isoleucine, valine) amino acids, but decreases in the presence of acidic (aspartic acid and glutamic acid) or sulfur-containing (cysteine and methionine) residues. Furthermore, it was shown that different families of peptides can be distinguished from the 2D plot [21]. It should be noted here that although this is an elegant approach, it has so only been used as a data representation method after de novo sequencing [21]. With the advent of modern high resolution (HR) mass spectrometry devices, it now becomes possible to use mass defect filtering of unknown peptides in complex samples.

In this study, we expand the mass mapping approach to select for cysteine-containing peptides prior to de novo sequencing, based on NMD, NIS, and overall mass. Although it is common practice to determine the monoisotopic mass of a peptide with high precision using modern high resolution mass spectrometers, the average mass is not a directly measurable value. The average mass can be calculated from the ion abundance of all detected isotopomers. We developed a data-dependent analysis workflow to experimentally determine the monoisotopic and average masses of unknown peptides, in order to calculate NIS and NMD values. By making a three-dimensional (3D) plot of NMD, NIS, and monoisotopic mass, peptides containing sulfur are differentiated from non-sulfur containing peptides, and those can then be further targeted for extensive MS^n^ based analysis and full primary structure elucidation. We demonstrate this workflow via the analysis of peptides in the secretions produced by the granular skin glands of two anuran amphibian species, *Odorrana schmackeri* and *Bombina variegata*.

## Experimental

### Granular Gland Skin Secretions

Skin secretion of *Odorrana schmackeri* and *Bombina variegata* were a kind gift from Professor Chris Shaw, Queen’s University, Belfast, UK. The crude lyophilized skin secretions (~100 μg) were resuspended in 100 μL of 50 mM ammonium bicarbonate containing 1 M urea and 25% acetonitrile. Insoluble material was removed by centrifugation at 17,800 relative centrifugal force (RCF) for 15 min. The supernatant was split in two parts; one part was directly measured by LC-MS and the other part was treated with 20 mM tris(2-carboxyethyl)phosphine (TCEP; Sigma-Aldrich, Munich, Germany) to reduce disulfide bonds.

### LC-MS

Samples were analyzed by on-line nanoflow LC-MS using an Agilent 1200 HPLC system and a LTQ-Orbitrap Velos (ThermoFisher Scientific, Bremen, Germany) mass spectrometer. Samples were injected onto an in-house made trap column with dimensions 20 mm L × 100 μm i.d., filled with particles of 5 μm diameter (Reprosil Pur C4; Dr. Maisch GmbH, Ammerbuch-Entringen, Germany). After trapping, the peptides were separated on an in-house made analytical column (C4, dimensions 140 mm L × 75 μm i.d., 5 μm particle size). The vented column setup was adjusted to an analytical column flow rate of 150–200 nL/min, solvent A was 0.6% acetic acid in Milli-Q, solvent B was 0.6% acetic acid in 80% acetonitrile in Milli-Q (v/v). The effluent of the column was directly sprayed into the ion source of the LTQ-Orbitrap Velos mass spectrometer. For determination of the monoisotopic and average mass, the most abundant multiply charged ions were automatically selected for HR zoom scans. Full Fourier transform MS scan width was set to the range 400–1500 *m/z* in profile mode with a resolution of 30,000 at 400 *m/z*. Multiply charged ions with a minimal intensity of 2 × 10^5^ were selected for a zoom scan with a resolution of 30,000 at 400 *m/z*, acquired in centroid mode with an isolation width set from –1.50 to +2.25 Dalton (Da) with respect to the selected precursor *m/z* value. For each zoom scan four microscans (AGC target value set to 1 × 10^5^, maximal injection time set to 500 ms) were combined. For the acquisition of HCD peptide fragmentation spectra, a second data-dependent analysis was performed. Fragmentation spectra were acquired at a resolution of 30,000 at *m/z* 400 starting with a fixed first mass at *m/z* 100. For each precursor, three separate HCD spectra were acquired at normalized HCD energy of 26, 28, and 30. The isolation window was set to 2.5 *m/z*, signal threshold for selection was 1 × 10^5^, AGC target was 5 × 10^5^, and maximum injection time was set to 200 ms.

### 3D Mass Mapping

The Xcalibur (ThermoFischer) *.raw files were converted to *.mzXML files using the ReAdW software (ver. 2.0, available at http://sourceforge.net/projects/sashimi/files/) and *.mzXML files were subsequently imported into the in-house developed MATLAB script (MATLAB and Bioinformatics Toolbox, release 2014a, The MathWorks, Inc., Natick, MA, USA). This script determines from each zoom scan spectrum the charge of the ion and calculates its monoisotopic mass. The average mass of each peptide is calculated from the peak height (ion intensity) and corresponding *m/z* values of all observed isotopomers above 3% in a zoom scan. This MATLAB script is available as Supplementary Material (Script-I).

For the determination of theoretical NMD and NIS values of all reviewed peptide/protein sequences from the animal venom and toxin annotation program [1, 2], all sequences smaller than 35 amino acids were downloaded in FASTA format, which allowed for direct import into MATLAB. From the primary amino acid sequence, the elemental composition was determined. Using this theoretical monoisotopic mass, nominal mass and average mass were calculated. The experimental nominal mass was calculated using Equation 1, and NIS and NMD values were calculated using Equations 2 and 3, respectively. For a reference see Artemenko et al. [21]. This MATLAB script is available as Supplementary Material (Script-II).

1 $$ \mathrm{Nominal}\;\mathrm{mass}=\mathrm{integer}\left(0.9995\times \mathrm{monoisotopic}\;\mathrm{mass}\right) $$

2 $$ \mathrm{N}\mathrm{I}\mathrm{S}=1000\times \left(\mathrm{average}\;\mathrm{mass}\hbox{--} \mathrm{monoisotopic}\;\mathrm{mass}\right)/\mathrm{monoisotopic}\;\mathrm{mass} $$

3 $$ \mathrm{N}\mathrm{M}\mathrm{D}=1000\times \left(\mathrm{monoisotopic}\;\mathrm{mass}\hbox{--} \mathrm{nominal}\;\mathrm{mass}\right)/\mathrm{monoisotopic}\;\mathrm{mass} $$

### Database Searching

Database searching of HCD fragmentation spectra acquired from both *O. schmackeri* and *B. variegata* was done using MASCOT (MASCOT server 2.2, Matrixscience Inc, Boston, MA, USA) against the UniProt entries anura (Tax ID 8342): protein and peptides sequences with 35 amino acids or less (downloaded in January 2015). The search parameters were set to the following; no enzyme, 5 ppm parent tolerance, 0.02 Da fragment tolerance, no enzyme specificity. C-terminal amidation was included as variable modification. De novo sequencing of selected peptides was done by combining information obtained by manual interpretation of the fragmentation spectra from different charge states of the peptide.

## Results and Discussion

**Figure 1:**
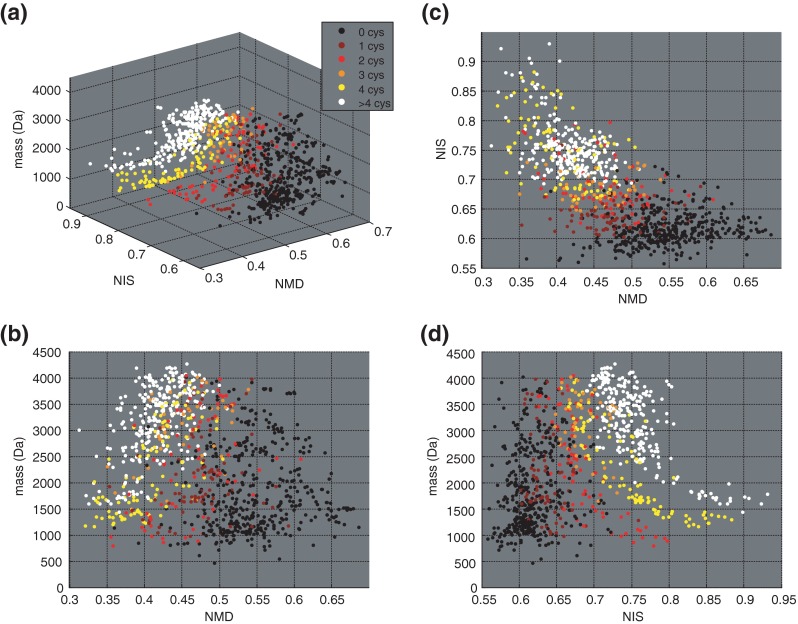
**(a)** Three-dimensional plot of NMD versus NIS versus monoisotopic mass (Da) of peptides smaller than 35 amino acids in UniProt animal venom and toxin database. Inset in **(a)** shows color scale used to label amount of cysteine residues in a sequence. **(b)** Two-dimensional plot (side view) of peptide mass versus NMD. **(c)** Side view of NIS versus NMD. **(d)** Side view of peptide mass versus NIS

Disulfide bridges are a common PTM encountered in animal toxin and venom peptides. To illustrate this, over 85% of all reviewed peptides in the UniProt Toxin and Venom database [1, 2] contain at least one disulfide bond. For all these peptide sequences up to 35 amino acids (a total of 1050 sequences), the mass, NIS and NMD were calculated (using Supplementary Information, Script-II) and plotted (Figure 1) using and a color code to differentiate the number of cysteines. A general trend is observed in which peptides with a molecular weight between 1000 and 2000 Da and no cysteines have low NIS (0.55–0.65) and high NMD values (0.50–0.75) (Figure 1a and d). Peptides with one or more cysteines shift to higher NIS and lower NMD values (Figure 1b), which is accompanied by increasing molecular weight. Based on the number of cysteines, distinct clusters can be discerned in the 3D plot in Figure 1. Using such a 3D mass map, threshold values for both NMD and NIS were inferred that can be used to select peptides for de novo sequencing.

Figure 1b–d illustrate the general trend that peptides with a NMD below 0.55, and a NIS higher than 0.65 are likely to contain cysteine residues. For some peptides with a high number of cysteines, NIS values can exceed 0.8 and NMD can even decrease to below 0.35. It should be noted here that besides cysteines, methionine residues contain a sulfur atom as well. Upon investigation of the amount of methionine residues present in this collection of sequences, it was established that peptides containing solely methionine as sulfur source also have a distinct localization in the plot, which differs from the cysteine-containing peptides (Supplementary Information, Figure I). This could due to the nature of the peptide (i.e., belonging to specific peptide families) or due to the fact that most methionine-containing peptides have only a single methionine-residue, whereas cysteine-containing peptides typically have multiple cysteine residues. As demonstrated for this collection of venom and toxin peptides, 3D mass mapping allows for filtering of cysteine-containing peptides from non-cysteine-containing peptides, even in cases where the latter contain a methionine residue. Our experimental determination of NMD and NIS values of unknown peptides is based on high resolution MS and zoom-scans. Determination of NIS and NMD values directly from the survey full scan MS spectrum would require a further advanced algorithm combining isotopic peak pattern and peak shape recognition. The use of a data-dependent MS^2^ based strategy based on zoom-scans works with much less complicated data processing.

**Figure 2:**
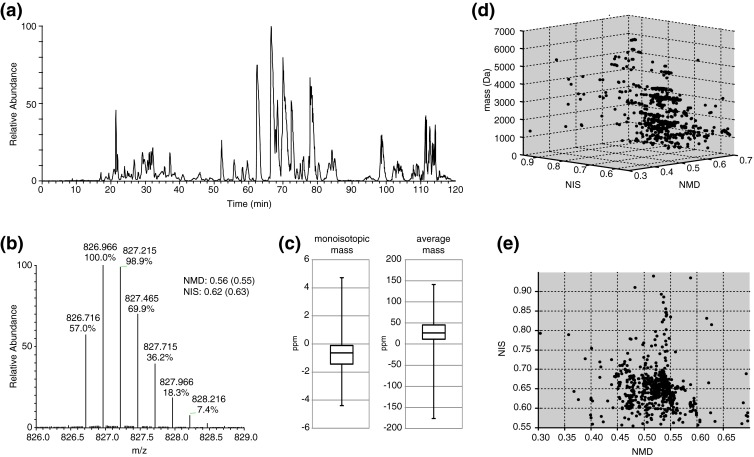
**(a)** Base peak intensity chromatogram of nanoflow HPLC-MS analysis of skin secretion of *Odorrana schmackeri.* **(b)** Zoom scan of [M + 4H]^4+^ of peptide Odorranain-C7HSa (UniProt accession: B4ERK5_ODOSH) with amino acid sequence SLLGTVKDLLIGAGKSAAQSVLKGLSCKLSKDC. Experimentally determined and theoretical values (in parenthesis) for NIS and NMD are given. **(c)** Box plots showing error distribution in the determination of monoisotopic and average mass (n = 104). **(d)** 3D plot of peptide mass, NIS, and NMD of unreduced *O. schmackeri*. **(e)** 2D plot of NIS and NMD

The skin secretion of *Odorrana schmackeri* is relatively well characterized and so far the primary structures of 27 peptides are listed in the UniProt protein database. The majority (25) of them have a C-terminal disulfide bond, often referred to as ‘Rana-box’ [22]. Skin secretion of *O. schmackeri* was reduced with TCEP and separated by nano-HPLC during a 120 min analysis. The base peak intensity chromatogram is depicted in Figure 2a. The MS system was programmed to acquire full scan spectra from *m/z* 400 to 1500. For each eluting multiply charged peptide exceeding the preset ion intensity threshold, a high resolution zoom-scan was acquired. A zoom-scan is the acquisition of the ion in a small *m/z* window that is written as a separate scan event in the raw data file. To avoid spectral contamination by other ions, an optimized zoom-scan window of –1.50 to +2.25 Da was used. Within the mass range of all peptides analyzed in this study and the multiple charging nature of electrospray ionization, all isotopomers above 1%–2% of a single peptide are visible within this window. The *.raw data-file was converted to an *.mzXML data-file which was imported into MATLAB, where all zoom scans were converted into a MATLAB structure. A script was programmed to process all zoom scans for calculation of both average and monoisotopic mass (Supplementary Information, Script-I).

The processing consists of several steps. First, all zoom scans of poor quality indicated by a low total ion count are discarded. For all remaining zoom scans, noise peaks are removed by discarding all intensity peaks below 3% of the most abundant peak. Using the remaining peaks, the charge state is determined, followed by calculation of average and monoisotopic mass. As an example, the most abundant peptide in the *O. schmackeri* skin secretion eluting at 68 min was selected. This peptide was identified as Odorranain-C7HSa (UniProt accession: B4ERK5_ODOSH) with amino acid sequence SLLGTVKDLLIGAGKSAAQSVLKGLSCKLSKDC, and Figure 2b shows the data-dependent zoom scan of the [M + 4H]^4+^. From the abundance of all isotopomers, the calculated NMD and NIS values are 0.56 and 0.62, respectively. These values are close to the theoretical values of 0.55 and 0.63, as calculated based on the chemical composition derived from the amino acid sequence.

To determine the accuracy of our workflow, a separate measurement was performed in which high resolution HCD fragmentation spectra were acquired. A MASCOT database search was performed against anura protein entries listed in the UniProt database (search results are provided in Supplementary Information, Table 1). A total of 35 protein identifications were made using 182 peptide spectral matches. Using the identified peptides, theoretical NIS and NMD values were calculated and compared with the experimental determined values. For a total of 104 peptides, we compared experimental monoisotopic mass and average mass with the theoretically calculated values (results are provided in Supplementary Information, Table 2). From this comparison, the error between experimental and theoretical monoisotopic mass and average mass were –0.73 and +26.2 ppm, respectively (see Figure 2c), indicating that our computation of average mass and subsequent calculation of NIS and NMD values are relatively accurate. Figure 2d and e show, respectively, the 3D plot of mass, NMD, and NIS and the 2D plot of NMD and NIS values for *O. schmackeri*. The majority of detected peptides have NMD values in the range of 0.45 to 0.50 and have NIS values in the range of 0.60 to 0.70, indicating a high proportion of disulfide bond containing peptides in this species’ skin secretion. This is also in agreement with the high number of disulfide bonds containing peptides identified in previous studies.

**Figure 3:**
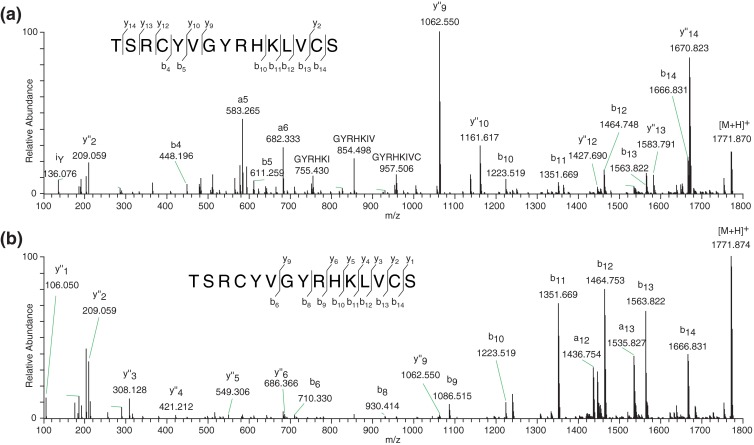
HCD fragmentation spectra of **(a)** [M + 4H]^4+^ at *m/z* 443.724 and **(b)** of the [M + 3H]^3+^ at *m/z* 591.297 of peptide TSRCYVGYRHK(I/L)VCS from *O. schmackeri* skin secretion

To further validate our approach, a peptide with a NMD of 0.49 and a NIS of 0.63, which had not been identified by the database search, was chosen for further primary structure elucidation. This was achieved through extended study of the fragmentation spectra of different charge states of the peptide’s precursor ion (see Figure 3 for the deconvoluted HCD fragmentation spectra of the peptide’s [M + 3H]^3+^ and [M + 4H]^4+^ ion). Interestingly, the two fragmentation spectra show relatively large differences in the pattern and degree of fragmentation. In the high mass-over-charge range the tandem MS spectrum of the 3+ ion shows a predominant series of b ions, whereas the fragmentation spectrum of the 4+ precursor reveals more prevalent y-ions in this region. In addition, the 4+ spectrum (Figure 3a) shows a relatively large degree of internal fragments around *m/z* 500–800, which are not seen for the 3+ spectrum (Figure 3b). Based on a combined analysis of both spectra a complete amino acid sequence of TSRCYVGYRHK[I/L]VCS is proposed, which was not possible from studying the individual fragmentation spectra. This underlines the difficulty of de novo sequencing non-tryptic peptides. A BLAST search against this sequence reveals a high sequence similarity of this peptide with the antimicrobial peptide Odorranain-T2-HN1 with amino acid sequence TSRCYVGYRRKIVCS (UniProt accession number: E7EKE4). The only difference between the newly identified *O. schmackeri* peptide and the earlier reported one from *O. hainanensis* is a histidine residue at position 10, instead of an arginine. It is therefore not unlikely that the newly discovered peptide has antimicrobial activity as well. This illustrates that even in a well-studied species, our 3D mass map screening approach helps to identify novel structures of potentially bioactive peptides.

**Figure 4:**
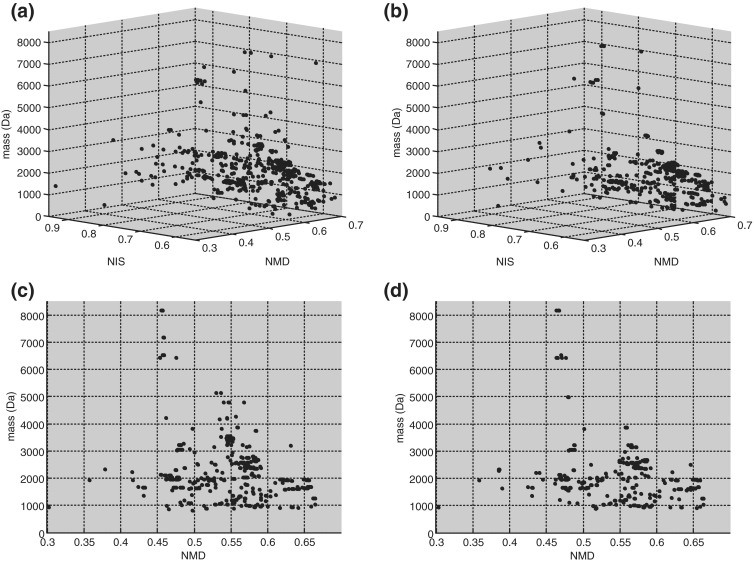
3D mass mapping results of unreduced and reduced skin secretion of *Bombina variegata*. Shown are 3D plot of mass, NMD, and NIS of **(a)** unreduced and **(b)** reduced peptides, and 2D plot of mass versus NMD of **(c)** unreduced and **(d)** reduced peptides

To explore the potential of our cysteine-containing peptide discovery strategy even further, the same workflow was applied to the skin secretion of *Bombina variegata*. From the skin of this particular frog, several peptides have been characterized previously, including the large family of antimicrobial bombinins and bombinin-like peptides [23, 24]. However, only a few frog skin peptides with one or more internal disulfide bonds have been described up till now (i.e., the insulin releasing peptides kininogen 1 and 2 [25], prokineticin [26], and the trypsin and thrombin inhibitor BSTI [27]. In Figure 4, the 3D and the 2D map of mass versus NMD are shown for both the reduced and the unreduced *B. variegata* sample. From this plot, several candidate peptides with low NIS and high NMD values were selected for a targeted MS^2^ analysis. Table 1 lists the results from this analysis, and many of the sequenced peptides have high similarity with kinogen 1 and 2 associated peptides. Also peptides with similarity to prokineticin and BSTI were found, although complete sequencing was not possible from the acquired spectra. Besides the peptides listed above, several novel structures were found and two of these will be discussed in more detail below.

**Table 1:** Cysteine-Containing Peptides Identified from the Skin Secretion of *B. variegata*

Monoisotopic mass (Da) ^a^	NMD	NIS	Number of SS-links	Amino acid sequence ^b, c^	Identity or similarity	Ref.
1557.732	0.47	0.66	1	DLCTFTSPGKVKCY-amide	Novel	
2111.971	0.46	0.68	1	ATHNMHWHRKPCNGPLLC	Similar to Kininogen-2-associated peptide	[28]
2272.856	0.38	0.65	1	FNQEDCFHEYLQNDAYCH	Novel	
2299.091	0.47	0.71	2	LYNALWPCKHCNKCKPGLLC	Similar to Kininogen-1-associated peptide	[25, 28]
2329.882	0.38	0.67	1	GFNQEDCFHEYIQNDAYCH	Novel	
2493.259	0.50	0.65	1	FKAPYNIHWHCKPGLLCKNIN	Similar to Kininogen-2-associated peptide	[28]
3034.483	0.48	0.66	1	LKYLYWPCKPGMPCENVD(898.477)	Kininogen-2-associated peptide	[28]
3803.893	0.50	0.65	1	LGSE(385.245)LKYLYWPCKPGM(1554.735)	Kininogen-2-associated peptide	[28]
3212.546	0.48	0.65	1	DMYELKGYKSAHGRPRVCPPGEQCPLWV-amide	Similar Bradykinin inhibitor peptide DV-28	[29]
3469.924	0.55	0.63	1	Chain 1: LQSLHKLRWPGKPLLLCENENGKLRPL-amide Chain 2: CLS	Novel	
3541.942	0.54	0.62	1	Chain 1: LQSLHKLRWPGKPLLLCENENEKLRPL-amide Chain 2: CLS	Novel	
6413.914	0.46	0.69	10	NFVCPPQGS(5494.6008)	Similar to BSTI	[27, 30]
6524.986	0.46	0.68	10	NFVCPPQGS(5605.6698)	Similar to BSTI	[27, 30]
7163.294	0.46	0.68	8	Chain 1: LKCNTLEGRGVQATLCPPGKETCMTHSVLLNGNTNLMKGCATFSRCS Chain 2: SVGSNRLSESLYCCNLNLCN	Novel	
8159.709	0.46	0.66	10	AVLTGT(374.138)DVQCGSGTCCATSVWS(5668.750)	Similar to Prokineticin Bv8	[26]

^a^ Measured monoisotopic masses of unreduced peptide

^b^ Sequences are given with all leucines; exact distinction between leucine and isoleucine could not be made

^c^ In cases where only partial sequence could be derived, remaining masses are given in parentheses

### Example I

**Figure 5:**
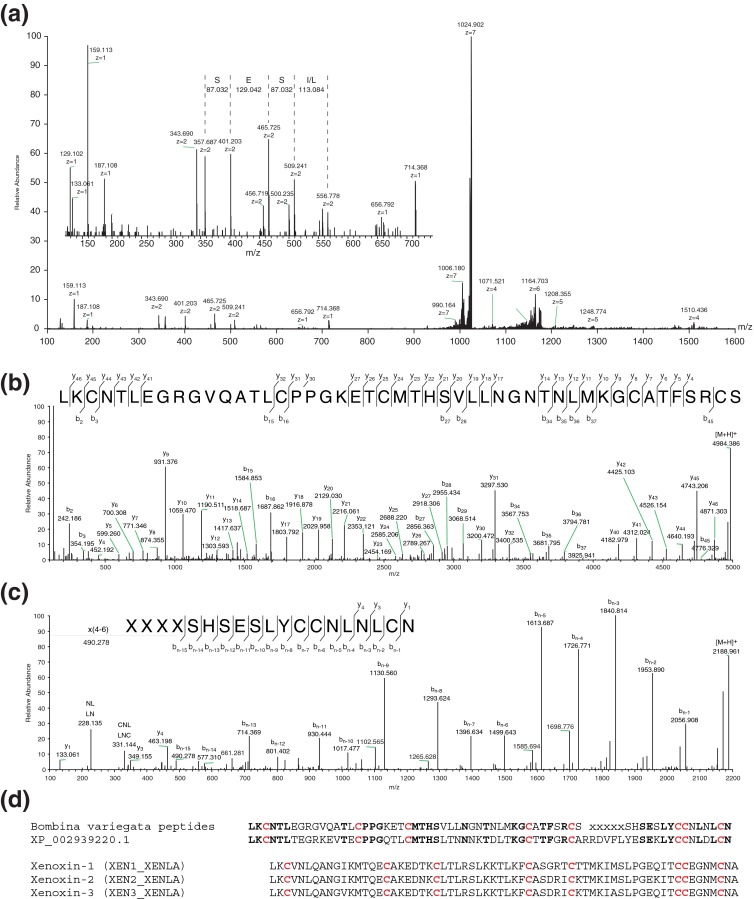
**(a)** HCD fragmentation spectra of [M + 7H]^7+^ at *m/z* 1024.332 of unreduced *B. variegata* peptide of monoisotopic mass 7163.273 Da. Inset shows short series of sequence ions. **(b)** HCD fragmentation spectrum of [M + 6H]^6+^ at *m/z* 831.572 of reduced *B. variegata* peptide of monoisotopic mass 4983.389 Da. **(c)** HCD fragmentation spectrum of [M + 3H]^3+^ at *m/z* 730.326 of reduced *B. variegata* peptide with monoisotopic mass of 2187.956. **(d)** Sequences of the *B. variegata* peptides, secreted Ly-6/uPAR-related protein 1-like from *X. tropicalis* (NCBI refseq: XP_002939220.1), and 3 xenoxins from skin secretion of *X. laevis*, with obvious similarities

One of the most peculiar examples is the peptide that has an apparent mass of 7163.273 Da (unreduced sample). This mass does not appear on the mass maps after reducing all the disulfide bonds in the sample. Consistently, two novel peptides with masses of 4983.389 and 2187.956 Da appeared in the reduced sample, both of them not present in the unreduced sample. The mass difference between single unreduced peptide and the two reduced (complementary) peptides is 8.071 Da, which corresponds to the mass increase upon reduction of four disulfide bonds. The HCD fragmentation spectrum of the large unreduced peptide (Figure 5a) shows a relatively low degree of fragmentation. From the fragmentation spectrum of the reduced peptide with molecular weight of 4983.389 Da (Figure 5b), a near complete sequence assignment could be made (except for distinction between isoleucine and leucine). From the fragmentation spectrum of the reduced peptide with molecular weight of 2187.956 Da (Figure 5c), a near complete sequence assignment could be made with only the 4–6 N-terminal residues unknown. More importantly, several of the sequence ions observed for this reduced peptide are identical to fragment ions seen in the fragmentation spectrum of the large unreduced peptide (inset of Figure 5a), confirming the unreduced peptide is a dimeric peptide of the two reduced peptides. Also, the number of identified cysteine residues (eight in total) confirms the expected presence of four disulfide bonds.

The carboxy-terminal end of the small peptide has strong similarity with the consensus sequence *CCXXXXCN* of 3-finger toxins, found in snake α-neurotoxins and frog cytotoxins [31, 32]. BLAST analysis of the two sequences against the NCBI nonredundant protein database shows highest sequence similarity to the secreted Ly-6/uPAR-related protein-1-like from *Xenopus tropicalis* (NCBI accession number: XP_002939220.1). For the larger chain, fragmentation at the last four C-terminal residues was not complete (Figure 5b). Based on the high similarity with the *X. tropicalis* sequence, a sequence assignment for the last four residues can be proposed. Figure 5d shows the sequences of the two *Bombina variegata* peptides and the Ly-6/uPar-related peptide and the xenoxins, cytotoxins identified from the dorsal gland secretions of *Xenopus laevis*. Besides identical positions for all the cysteines, the sequences of *B variegata* and *X. tropicali*s show several other conserved residues. The sequence of *X. tropicalis* originates from DNA sequencing and evidence at protein level or function is not known yet. The similarity with the xenoxins previously shown to be present in the skin secretion of *X. laevis* [33, 34] is much lower (Figure 5d). Interestingly, the prototype and other described members of this family of 3-finger toxin proteins are all known to be composed of a single polypeptide chain. Remarkably the *B. variegata* variant discovered here is composed of two chains, forming an intermolecular S–S-linked heterodimer. Most likely this heterodimer is synthesized by processing of a single polypeptide chain. Reanalysis of the MS and MS^2^ data did not show evidence for the presence of this full length single polypeptide and it also did not yield in the detection of other heterodimeric forms of this peptide, suggesting that the observed heterodimer is specifically formed and not the result of specific degradation.

### Example II

**Figure 6:**
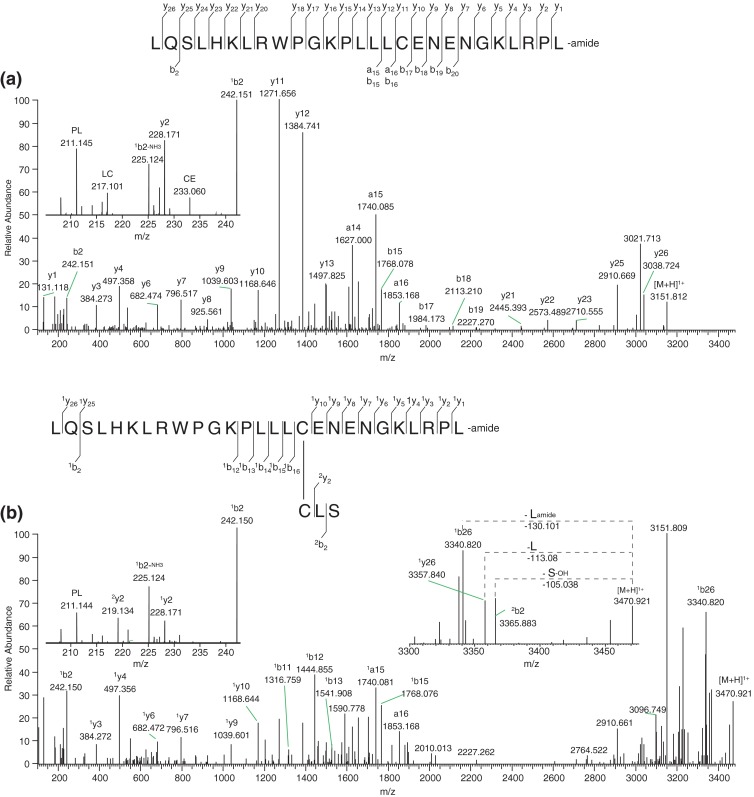
HCD fragmentation spectra of heterodimeric disulfide bond linked peptide from *B. variegata* with molecular mass of 3469.924 Da. **(a)** Deconvoluted HCD spectrum of the large reduced chain, ([M + 6H]^6+^ at *m/z* 526.141 (monoisotopic peak) was selected for fragmentation). **(b)** Deconvoluted HCD spectrum of unreduced dimeric peptide, ([M + 6H]^6+^ at *m/z* 579.329 (monoisotopic peak) was selected for fragmentation. Insets highlight the loss of 1 N-terminal and 2 C-terminal residues

We discovered two more novel heterodimeric *B. variegata* peptides (with masses of 3469.924 and 3541.942 Da). The sequences of these two homologous dimeric peptides are, respectively, (*LQSLHKLRWPGKPLLLCENENGKLRPLamide-CLS* and *LQSLHKLRWPGKPLLLCENENEKLRPLamide-CLS)* (Table 1). The sequences are given with all leucines*,* but distinction between isoleucine and leucine could not be made. Figure 6 shows the fragmentation spectra of the reduced and unreduced form of the peptide with mass 3469.924 Da. The spectra for the peptide with mass 3541.942 Da are given in Supplementary Figure 2. Both dimeric peptides consist of a chain with 27 amino acids, which is amidated at the C-terminal residue. They only differ from each other with either a glutamic acid or glycine at position 22. By analysis of the HCD fragmentation spectra of the charge states 4+ to 6+, a complete sequence assignment could be made (except the I/L distinguishing) and Figure 6a shows the HCD fragmentation spectrum with a, b, and y ion annotation of the 5+ charge state. The other chain of both heterodimers is identical for both and consists of only three amino acids. This chain was not recovered in the analysis of the reduced sample, but the fragmentation spectra of both unreduced dimers evidently showed that in the small chain the Cys is the N-terminal amino acid flanked by an Ile/Leu and Ser at the C-terminus (Figure 6b and Supplementary Figure 2B). This was deduced from the loss of three single amino acids from the precursor ion, which included the C-terminal amidated Ile/Leu of the large chain, and the losses of a C-terminal Ser. In the low *m/z* region, in the reduced peptide, internal fragments corresponding to CysGlu and (ILe/Leu)Cys were observed, whch were not observed for the unreduced peptide. Simultaneously, for the unreduced dimeric peptide, a y2 ion at *m/z* 219.134 was observed, corresponding to the two C-terminal amino acids, (Ile/Leu)Ser of the small chain. The two latter observations prove that both peptides are indeed a disulfide bond-linked dimeric peptide. BLAST analysis against anura sequences in the NCBInr database showed no significant similarity with any other known frog skin peptide, indicating this is a novel structure.

## Conclusions

The normalized nominal mass defect and normalized isotope shift of peptides containing cysteine residues renders them a specific spatial distribution in 2D and 3D mass maps. We developed a MATLAB script to compute both values from experimentally obtained high resolution (orbitrap) mass spectrometry data. Together with 3D and 2D mass mapping, the script considerably enhances the efficiency with which disulfide bridge containing peptides can be detected in complex biological samples, such as amphibian skin defensive secretions, and can be considered as a tool to discover peptides that require this structural element for biological activity and stability. After illustrating the concept by analyzing a subset of the UniProt database representing a collection of animal toxin sequences, we validated our approach experimentally on the skin secretion of two frog species. One of these species (*O. schmackeri*) is known to contain many such S–S linked peptides; the other (*B. variegata*) is much less studied in this respect. The workflow we elaborated indeed selects previously reported peptides with intramolecular S–S bonds. In addition, from *B. variegata* skin secretion novel intra- and inter-molecular disulfide bridge-forming peptides were discovered, confirming the usefulness of this novel approach.

## Electronic supplementary material

Supplementary Figure 1 **(A)** Three dimensional plot of NMD versus NIS versus mass (Da) of peptides smaller than 35 amino acids in the ToxProt database. The legend shows the color scale used to indicate the number of cysteine and methionine residues. **(B)** Side view of NMD versus NIS, **(C)** side view of NMD versus peptide mass and **(D)** side view of NIS versus mass of peptide. (PDF 222 kb)

Supplementary Figure 2 HCD fragmentation spectra of a heterodimeric disulfide bond linked peptide from the skin secretion of *Bombina variegata* with molecular mass of 3541.942 Da. **(A)** deconvoluted HCD spectrum of the large chain, selected for fragmentation was the [M+6H]^6+^ at *m/z* 538.144 (monoisotopic peak). **(B)** deconvoluted HCD spectrum of the dimeric unreduced peptide, selected for fragmentation was the [M+6H]^6+^ at *m/z* 591.332 (monoisotopic peak). Insets highlight the loss of 1 N-terminal and 2 C-terminal residues. (PDF 26 kb)

Supplementary Table 1&2 Excel file with Mascot search summary for *Odorrana schmackeri* skin secretory peptides identified **(Table 1)** and a comparison of experimental and theoretical NIS and NMD values **(Table 2)**. (XLS 446 kb)

Supplementary material 1 MATLAB file and accompanying PDF document file with Script-I for the calculation of experimental monoisotopic, average and nominal mass and NIS and NMD values from an mzXML file containing zoom-scans. (M 19 kb) (PDF 424 kb)

Supplementary material 2 MATLAB file and accompanying PDF document with Script-II for the calculation of theoretical monoisotopic, average and nominal mass and NIS and NMD values from a FASTA-file containing peptide/protein sequences. (M 8 kb) (PDF 372 kb)
