# Supplementary figures and images for: Screening Method for the Discovery of Potential Bioactive Cysteine-Containing Peptides Using 3D Mass Mapping
Source: J Am Soc Mass Spectrom. 2015 Nov 9;26(12):2039–50. doi: 10.1007/s13361-015-1282-z (PMC4654750; doi:10.1007/s13361-015-1282-z)

Supplementary figure 1

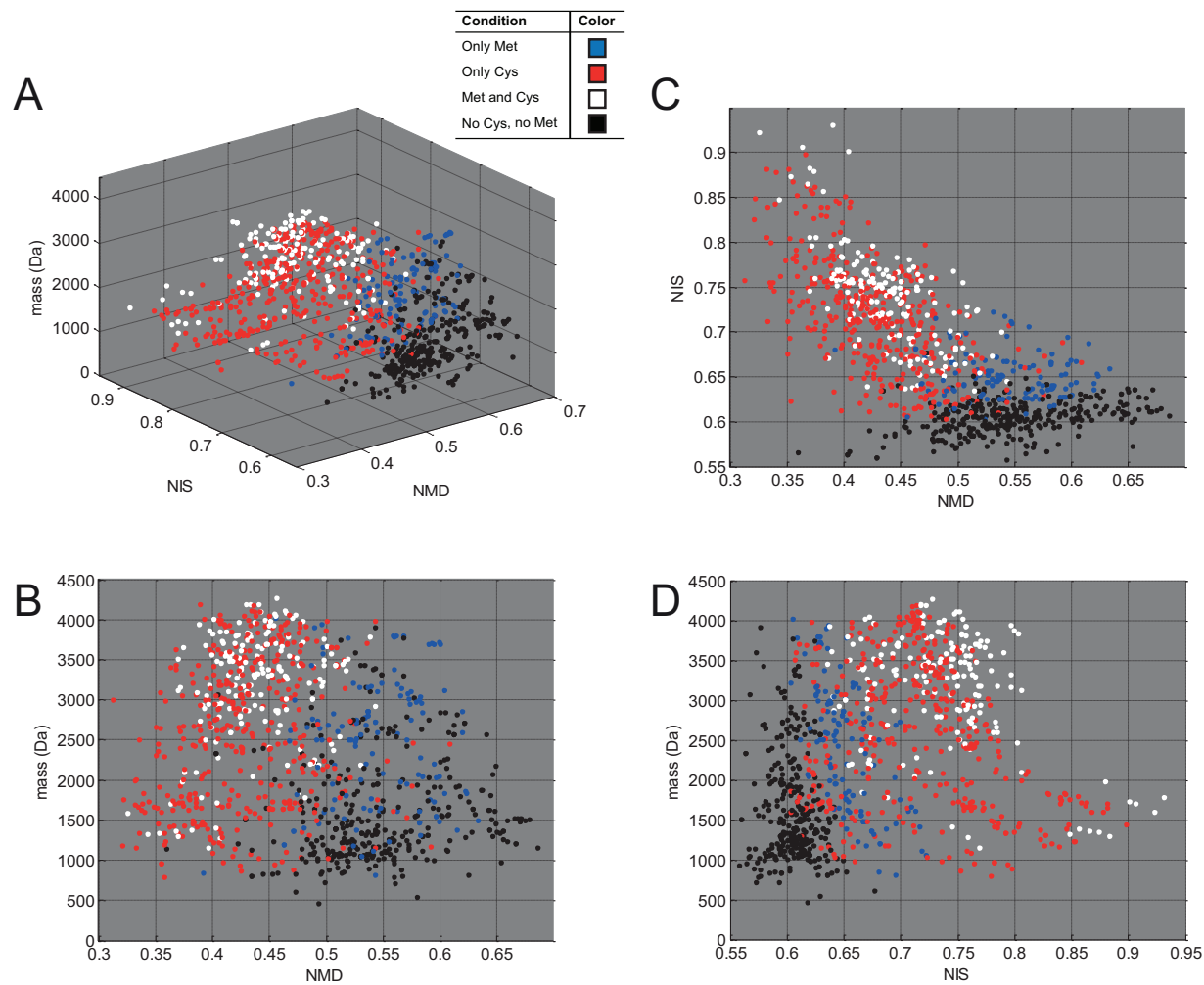

Supplement: Supplementary file 1 — (A) Three dimensional plot of NMD versus NIS versus mass (Da) of peptides smaller than 35 amino acids in the ToxProt database. The legend shows the color scale used to indicate the number of cysteine and methionine residues. (B) Side view of NMD versus NIS, (C) side view of NMD versus peptide mass and (D) side view of NIS versus mass of peptide. (PDF 222 kb) [file 13361_2015_1282_MOESM1_ESM.pdf]

Supplementary Figure 2

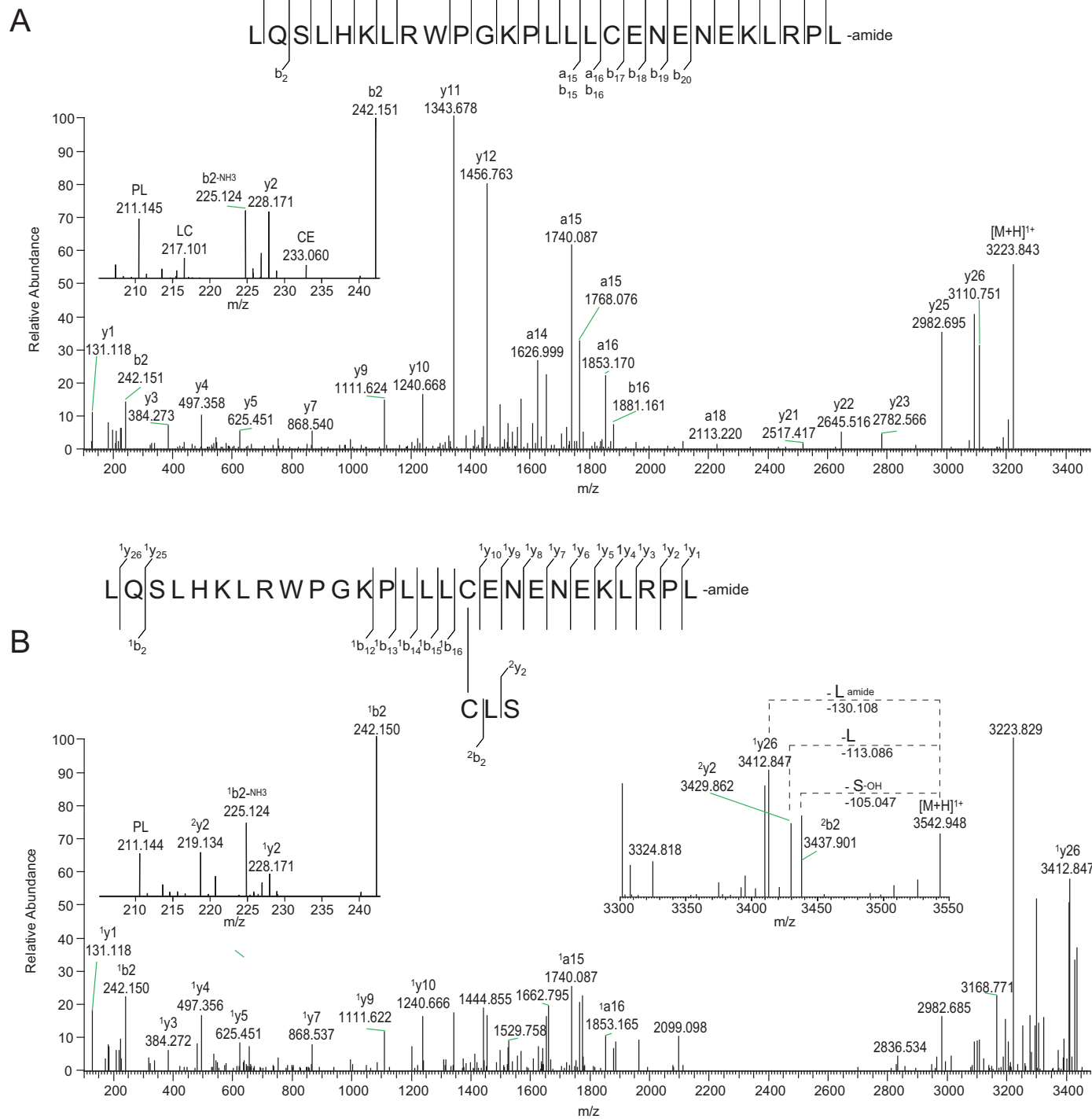

Supplement: Supplementary file 2 — HCD fragmentation spectra of a heterodimeric disulfide bond linked peptide from the skin secretion of Bombina variegata with molecular mass of 3541.942 Da. (A) deconvoluted HCD spectrum of the large chain, selected for fragmentation was the [M+6H]6+ at m/z 538.144 (monoisotopic peak). (B) deconvoluted HCD spectrum of the dimeric unreduced peptide, selected for fragmentation was the [M+6H]6+ at m/z 591.332 (monoisotopic peak). Insets highlight the loss of 1 N-terminal and 2 C-terminal residues. (PDF 26 kb) [file 13361_2015_1282_MOESM2_ESM.pdf]
