# Supplementary material for: Screening Method for the Discovery of Potential Bioactive Cysteine-Containing Peptides Using 3D Mass Mapping
Source: J Am Soc Mass Spectrom. 2015 Nov 9;26(12):2039–50. doi: 10.1007/s13361-015-1282-z (PMC4654750; doi:10.1007/s13361-015-1282-z)
Supplement: Supplementary file 5 — (PDF 424 kb) [file 13361_2015_1282_MOESM5_ESM.pdf]

Script to process mzXML file 1
Erase all temporary files 2
Define required information and define variables 2
Import mzXML to a MATLAB structure 3
Add ScanType to 'peaklist' 3
Extract scan number 3
Extract zoom scans. 4
Delete all spectra based on highest intensity peak 4
Delete zoom scans were only one peak is present 4
Remove noise from spectra 5
Delete zoom scans were only one peak is present 6
Calculate charge and monoisotopic mass 6
Calculate mass of isotopomers 7
Calculate the mass difference between isotopomers 7
Calculate average mass 8
Add scan number to data matrix 8
Data generation for 3D plot 9
Remove miscalculated NMD values 9
Remove miscalculated NIS values 10
Add fraction number to matrix 'mass3' 11
Generate the 3D mass map 11
3D mass map properties 11
Set view of 3D mass map 12
Export table 'mass3' to an EXCEL file 12

Script to process mzXML file

```
% The following script accompanies the article "Screening method for the
% discovery of potential bioactive cysteine-containing peptides using 3D
% mass mapping" and in particular Figure 2D, 2E, 4A, 4B, 4C and 4D.

% In this script, mzXML-files containing raw MS data are imported into
% a MATLAB structure for further analysis. The mzXML-files were generated
% from Thermo *.raw-files using the ReAdW software (version 2.0,
% publicly available at
% <http://sourceforge.net/projects/sashimi/files/>. Subsequent analysis
% of the data was done using MATLAB and the Bioinformatics Toolbox,
% version 8.3.0.532 (R2014a) on a Microsoft Windows 8 Version 6.2 (Build
% 9200) operating system. The version of Java installed was 1.7.0_11-b21
% with Oracle Corporation Java HotSpot(TM) 64-Bit Server VM, mixed mode.
```

```
% From the MATLAB structure generated, the zoom scan events are extracted.
% From each zoom scan spectrum the monoisotopic and average mass is
% calculated from the observed isotopomers. Using the monoisotopic mass and
% the average mass the nominal mass, NMD and NIS (equation 1, 2 and 3 in
% the corresponding article respectively) are calculated. Subsequently a
% three-dimensional plot is generated (NMD vs NIs vs monoisotopic mass) as
% described in the article. Corresponding data from the LC-MS run is
% exported into an EXCEL-file (*.xlsx extension).

% Disclaimer: the authors would like to state that this script has been
% used only with data obtained using an LTQ-Orbitrap Velos mass
% spectrometer (Thermo Scientific).

% For questions, clarification, tips or feedback try to seek
% contact through the MATLAB File Exchange
% (http://www.mathworks.com/matlabcentral/fileexchange/) or contact the
% authors of the corresponding article directly.
```

Erase all temporary files

```
% Clear the workspace, close all windows and clear the command-window.

clear all
close all
clc
```

Define required information and define variables

```
% Raw data was generated using an Thermo LTQ velos orbitrap. The resulting
% Thermo *.raw files were converted to *.mzXML files using the ReAdW
% software.
% Make sure that the mzXML file is conform the mzXML 2.1 specifications or
% EARLIER versioins, otherwise the matlab function 'mzxmlread' will not be
% able to process the mzXML file.

% Samples may be fractionated before LC-MS analysis. In that case it is
% helpful to indicate in the final *.xlsx file the fraction number, as well
% as a proper title (For example the same title of the original *.mzxml
% file).

% In this script, zoom scans are extracted based on intensity of the
% most intense ion as a threshold. It was found (using trial and error)
% that an ion count of 50,000 for the highest peak per spectrum
% corresponded to high quality zoom scan spectra. The value can be adjusted
% here accordingly

% To remove noise from the zoom scan spectra, all peaks with an intensity
% below 3% of the most intense peak are discarded. This percentage can be
% adjusted in this section

% specify the mzXML data file.
datafile = 'your_mzxmlfile.mzxml';

% specify fraction number.
Fraction_nr = 1;
```

```
% specify title for exported *.xlsx file.
title = 'filename_fraction_nr';

% specify threshold intensity value for highest peak.
highestpeak = 50000;

% specify noise removal threshold.
percentage = 0.03;

% specify mass of proton.
mass_proton = 1.00727;
```

Import mzXML to a MATLAB structure

```
% Build the mzXML structure using 'mzxmlread' from the bioinformatics
% toolbox.
mzxml_struct = mzxmlread(datafile);

% Store all scans in cell array 'peaklist'. Each scan is a matrix inside
% the cell array. The first column of this matrix indicates m/z values, the
% second column the ion intensities of that corresponding m/z value.
[peaklist] = mzxml2peaks(mzxml_struct, 'Levels', [1;2]);
```

Add ScanType to 'peaklist'

```
% Indicates which scan events are of the type 'Full' (the case for full and
% MS2 scan events) or 'SIM' (selected ion monitoring; the zoom scan events).

%  loop over the length of the peaklist (containing all scan events), and
%  add the scan type (as a string) in the next column of cell array 'peaklist'.
for i=1:length(peaklist)

    % Generate the cell array 'scanType' fom mzXML_struct.scan.scanType.
    % This cell array 'scanType' holds the information (Full or SIM) in the
    % first column as a string.
    scanType = {mzxml_struct.scan.scanType}';

    % Copy the first column of the cell array 'scanType' to the second column
    % of the cell array 'peaklist'.
    peaklist{i,2} = scanType{i,1};

end % close the loop
```

Extract scan number

```
% To keep track of which scan (with corresponding scan type) corresponds to
% which scan number in the original *.raw file, the scan number is added to
% the third column of the cell array 'peaklist'.

% loop over the length of 'peaklist' and add the scan number.
for i=1:length(peaklist)

    % Create a new cell array called 'num' from mzXML_struct.scan.num. This
```

```
    % cell array 'num' holds the scan numbers in the first column
    num = {mzxml_struct.scan.num}';

    % copy the first column of the array 'num' to the third column of the
    % array 'peaklist'.
    peaklist{i,3} = num{i,1};

end % close the loop
```

Extract zoom scans.

```
% Create cell array 'peaklist_SIM' containing only SIM scan (zoom scan) events by
% searching 'peaklist' for the exact match 'SIM'.
peaklist_SIM = peaklist(strmatch('SIM', peaklist(:,2), 'exact'),1:3);
```

Delete all spectra based on highest intensity peak

```
% Zoom spectra of poor quality (indicated by low intensity peaks) are
% discarded on basis of the intensity of the highest peak in that spectrum.
% The threshold value is set in the second section under 'highestpeak'.
% Remaining scans are stored in 'peaklist_Q'.

% start to write at row 1 when storing information in 'peaklist_Q'.
row = 1;

% loop over the length of peaklist_SIM (which contains all zoom scan
% events).
for i=1:length(peaklist_SIM)

    % Check if the maximum ion intensity in each scan event is higher than
    % the set threshold of 'highestpeak'
    if max(peaklist_SIM{i,1}(:,2)) > highestpeak

    % preserve relevant accompanying info of spectrum.
    peaklist_Q{row,1} = peaklist_SIM{i,1};
    peaklist_Q{row,2} = peaklist_SIM{i,2};
    peaklist_Q{row,3} = peaklist_SIM{i,3};

    %  move to next row.
    row = row + 1;

    end % close the loop

end % close the loop
```

Delete zoom scans were only one peak is present

```
% Spectra with only one peak are removed. Remaining scan events
% are stored in 'peaklist_Q2'.

% start to write at row 1 when storing information in 'peaklist_Q2'.
row = 1;

% loop over the length of peaklist_Q (containing all scan events with a
```

```
% peak over 50,000 counts).
for i=1:length(peaklist_Q);

    % Check if the amount of remaining peaks in a spectrum-matrix are more
    % than 1.
    if length(peaklist_Q{i,1}(:,1)) >= 2

        % preserve relevant accompanying info of the spectrum
        peaklist_Q2{row,1} = peaklist_Q{i,1};
        peaklist_Q2{row,2} = peaklist_Q{i,2};
        peaklist_Q2{row,3} = peaklist_Q{i,3};

        % move to the next row.
        row = row + 1;

    end % close the loop

end % close the loop
```

Remove noise from spectra

```
% remove noise peaks with an intensity below 3% of the maximum intensity.

% start to write at row 1 when storing information in 'peaklist_denoised'.
row = 1;

% loop over the length of peaklist_Q2 (containing all spectra with more
% than one peak and one peak over 50,000 counts).
for i=1:length(peaklist_Q2)

        % start to count at 1.
        count = 1;

        % Calculate threshold ion intensity as percentage of the maximum
        % intenstiy per spectrum.
        threshold = max(peaklist_Q2{i,1}(:,2)) * percentage;

        % loop over the length of peaklist_Q2.
        for j = 1:length(peaklist_Q2{i,1})

            if peaklist_Q2{i,1}(j,2) > threshold

            % preserve relevant accompanying info per spectrum
            peaklist_denoised{row,1}{count,1} = peaklist_Q2{i,1}(j,1);
            peaklist_denoised{row,1}{count,2} = peaklist_Q2{i,1}(j,2);
            peaklist_denoised{row,2} = peaklist_Q2{i,2};
            peaklist_denoised{row,3} = peaklist_Q2{i,3};
            count = count + 1;

            end % close the loop

        end % close the loop

        % move to the next row.
        row = row +1;
```

```
end % close the loop
```

Delete zoom scans were only one peak is present

```
% Spectra with one remaining peak are removed

% start to write at row 1 when storing information in 'peaklist_denoised2'.
row = 1;

% loop over peaklist_denoised.
for i=1:length(peaklist_denoised)

    % Check if the amount of remaining peaks in a spectrum-matrix are more
    % than 1.
    if length(peaklist_denoised{i,1}(:,1)) >= 2

       % preserve relevant accompanying info per spectrum
       peaklist_denoised2{row,1} = peaklist_denoised{i,1};
       peaklist_denoised2{row,2} = peaklist_denoised{i,2};
       peaklist_denoised2{row,3} = peaklist_denoised{i,3};

       % move to next row.
       row = row+1;

    end % close the loop.

end % close the loop.
```

Calculate charge and monoisotopic mass

```
% The m/z values are sorted descending. The charge (z) and the monoisotopic
% mass of the peptide are calculated by z = 1/( m/z1 - m/z2), where m/z1
% and m/2 are adjecent isotopomers in a spectrum.

% loop over peaklist_denoised2.
for i=1:length(peaklist_denoised2);

    % sort ion intensities descending
    peaklist_denoised2{i,1} = sortrows(peaklist_denoised2{i,1},-2);

    % calculate charge (z).
    z = abs(round((1/(peaklist_denoised2{i,1}{1,1} - peaklist_denoised2{i,1}{2,1}))));

        % loop over peaklist_denoised2.
        for j=1:length(peaklist_denoised2{i,1}(:,1));

        % store charge (z) in column 3 of the matrix describing the
        % scan event.
        peaklist_denoised2{i,1}{j,3} = z;

        end % close the loop

            % Sort m/z values in a scan ascending.
            peaklist_denoised2{i,1} = sortrows(peaklist_denoised2{i,1},1);
```

```
            % calculatemonoisotopic mass ('Mass_peptide')
            % and mass of isotopomers in a spectrum by
            % using m/z value and charge. Store this value in the
            % 4th column of 'peaklist_denoised2'.

                % loop over peaklist_denoised2.
                for j=1:length(peaklist_denoised2{i,1}(:,1));

                % store the mass of the peptide in the 4th column of the
                % matrix describing the scan event
                m_z = peaklist_denoised2{i,1}{j,1};
                Mass_peptide = (m_z * z) - (z * mass_proton);
                peaklist_denoised2{i,1}{j,4}= Mass_peptide;

                end  % close the loop

end % close the loop
```

Calculate mass of isotopomers

```
% To calculate the average mass, the mass of each isotopomer in a
% zoom scan spectrum is multiplied by its ion intensity. These values, the
% 'weighted ions' are stored in the fifth column of a spectrum matrix.
% the sum of all weighted ions is devided by the sum of all the ion counts
% to yield the average mass.

% Loop over the length of 'peaklist_denoised2'.
for i=1:length(peaklist_denoised2)

    % Loop over the length of the matrix describing the scan event.
    for j=1:length(peaklist_denoised2{i,1}(:,1))

        % calculate all weighted ions. Store in column five of a spectrum matrix.
        peaklist_denoised2{i,1}{j,5}= peaklist_denoised2{i,1}{j,4} *
peaklist_denoised2{i,1}{j,2};

    end % close the loop

end % close the loop
```

Calculate the mass difference between isotopomers

```
% It is possible that besides the protonated form, the same peptides with
% adducts (ammonia, potassium, sodium, etc) or the oxidized form of the
% corresponding peptide appears within the same zoom scan. Even with a
% narrow zoom scan window, this can occur for highly charged (>7+) ions.
% To discard spectra that contain these interfering peaks of
% adducts or oxidized residues, the mass difference between the last and
% first isotopomer in a zoom scan spectrum is calculated. If this mass
% difference is above 14 Da, the spectrum is discarded.

% Loop over the length of 'peaklist_denoised2'.
for i=1:length(peaklist_denoised2)
```

```
    % calculate the difference between the first and last mass of the
    % present isotopomers in a spectrum
    peaklist_denoised2{i,1}{1,6} = (peaklist_denoised2{i,1}{end,4}) -
(peaklist_denoised2{i,1}{1,4});

end % close the loop

% Start to write at row 1 when storing preserved spectra
row = 1;

% Loop over the length of 'peaklist_denoised2'.
for i=1:length(peaklist_denoised2)

    % check if the mass difference in a spectrum between the first and last
    % isotopomer is smaller than 14 Da.
    if peaklist_denoised2{i,1}{1,6} < 14

        % preserve relevant information
        peaklist_denoised3{row,1} = peaklist_denoised2{i,1};
        peaklist_denoised3{row,2} = peaklist_denoised2{i,3};

        % move to the next row
        row = row+1;

    end % close the loop

end % close the loop
```

Calculate average mass

```
% Loop over the length of 'peaklist_denoised3'
for i=1:length(peaklist_denoised3)

    % Sum all ion counts (column 2 in a scan matrix)
    sum_ion = sum(cell2mat(peaklist_denoised3{i,1}(:,2)));

    % Sum all 'weighted ions' (column 5 in a scan matrix).
    sum_weighion = sum(cell2mat(peaklist_denoised3{i,1}(:,5)));

    % Divide sum of 'weighted ions' by sum 'ions', resulting in the 'average mass'.
    % store average mass in matrix 'mass' (column 2)
    mass(i,2)=sum_weighion/sum_ion;

    % store the monoisotopic mass in the matrix 'mass' (column 1)
    mass(i,1)=peaklist_denoised3{i,1}{1,4};

end % close the loop
```

Add scan number to data matrix

```
% Loop over the length of peaklist_denoised2
for i=1:length(peaklist_denoised2)

    % Add scan number in column 3 of matrix 'mass'
    mass(i,3)= peaklist_denoised2{i,3};
```

```
end % close the loop
```

Data generation for 3D plot

```
% Using the formula's given in the corresponding article, calculate nominal
% mass, NMD and NIS.

% loop over the length of 'mass'
for i=1:length(mass)

    % Store the nominal mass in the fourth column of matrix 'mass'.
    mass(i,4) = round(     double(((mass(i,1)*0.9995))));

end % close the loop

% One has now: the monoisotopic mass (mass(i,1)), average mass (mass(i,2))
% and nominal mass (mass(i,4)) and the corresponding scannumber in
% (mass(i,3)).



% loop over the length of 'mass'
for i=1:length(mass)

    % Calculate the NMD and NIS and store in matrix mass(i,5) and mass(i,6)
    % respectively.
    mass(i,5) = 1000 * (  mass(i,1)-mass(i,4) ) / mass(i,1);
    mass(i,6) = 1000 * (  mass(i,2)-mass(i,1) ) / mass(i,1);

end % close the loop

% loop over the length of 'mass'
for i=1:length(mass)

    % Put in the table 'mass' the first isotopomer (m/z peak) in column 7
    mass(i,7) = peaklist_denoised2{i,1}{1,1};

end % close the loop
```

Remove miscalculated NMD values

```
% filter on NMD values (mass(i,5)). The NMD value must be between 0 and 1.
% These limits are defined as X1 (0) and X2 (1).

% Set limits
X1 = 0;
X2 = 1;

% Start to write at row one when storing information
row = 1;

% loop over the length of 'mass'
for i=1:length(mass)
```

```
    % Keep scans that have NMD between X2 and X1.
    if mass(i,5) <= X2 && mass(i,5) >= X1;

        % Preserve relevant data.
        mass2(row,1) = mass(i,1);
        mass2(row,2) = mass(i,2);
        mass2(row,3) = mass(i,3);
        mass2(row,4) = mass(i,4);
        mass2(row,5) = mass(i,5);
        mass2(row,6) = mass(i,6);
        mass2(row,7) = mass(i,7);

        % move to the next row
        row = row+1;

    end % close the loop

end % close the loop
```

Remove miscalculated NIS values

```
% filter on NIS values (mass(i,6)). The NMD value must be between 0 and 1.
% These limits are defined as Y1 (0) and Y2 (1).

% Set limits
Y1 = 0;
Y2 = 1;

% Start to write at row one when storing information
row = 1;

% loop over the length of 'mass'
for i=1:length(mass2)

    % Keep scans that have NMD between X2 and X1.
    if mass2(i,6) <= Y2 && mass2(i,6) >= Y1;
       mass3(row,1) = mass2(i,1);
       mass3(row,2) = mass2(i,2);
       mass3(row,3) = mass2(i,3);
       mass3(row,4) = mass2(i,4);
       mass3(row,5) = mass2(i,5);
       mass3(row,6) = mass2(i,6);
       mass3(row,7) = mass2(i,7);

       % add charge to row 8
       mass3(row,8) = round(mass2(i,2)/mass2(i,7));

       % move to the next row
       row = row+1;

    end % close the loop

end % close the loop
```

Add fraction number to matrix 'mass3'

```
mass3(:,9) = Fraction_nr;
```

Generate the 3D mass map

```
% make a 3D scatterplot using scatter3(x,y,z,s,t). the
% values for x, y and z are corresponding to NMD, NIS and MASS and
% the 's' is the size (area) of each marker as determined in the vector s.
% The 't' is the color of the marker.


%NMD
x = mass3(:,5);
%NIS
y = mass3(:,6);
%MASS
z = mass3(:,1);

s = 15;
t = 'k';

% Make the plot

 plot = scatter3(x, y, z, s, t, 'fill','MarkerEdgeColor',[0 0 0]); hold on
```

3D mass map properties

```
% Get the handle to the figure and set the background color to white.
set(gcf, 'color', [1 1 1])

% Get the handle to the current axis and set the color of the background
% to a shade of grey.
set(gca, 'color', [0.4 0.4 0.4])

% Get the handle to the current axis and set the color of all three
% axis to black.
set(gca, 'XColor', 'k')
set(gca, 'YColor', 'k')
set(gca, 'ZColor', 'k')


% Label the x, y and z axis accordingly. The default fontsize of the labels
% is 10. Set to size 12.
xlabel('NMD', 'FontSize', 12);
ylabel('NIS', 'FontSize', 12);
zlabel('mass (Da)', 'FontSize', 12);

% get the handle to the current axis and set fontsize to 12.
set(gca, 'FontSize', 12)

% set axis min and max values using 'axis([ xmin xmax ymin ymax zmin zmax])
% depending on the NMD (x), NIS (y) and mass (z) of peptides in the mzXML
% file that was processed. In general NMD ranges from 0.3 to 0.7 and NIS
% ranges from 0.55 to 0.95. The mass of the peptides can vary and thus be
```

```
% set accordingly here. To include all peptides one can use max(mass3(:,1))
axis([0.3 0.7 0.55 0.95 0 max(mass3(:,1))]);
```

Set view of 3D mass map

```
% default view is 'view(3)'. Some 2D representational views are
% listed below.

% % For 2D view of NMD vs NIS, use:
% view([0 0 1])

% % For 2D view of NMD vs Mass, use:
% view([0 -1 0])

% % For 2D view of NIS vs Mass, use:
% view([1 0 0])
```

Export table 'mass3' to an EXCEL file

```
% Upon creating a new sheet in a new EXCEL file, MATLAB throws a warning.
% disable the warning
warning('off','MATLAB:xlswrite:AddSheet')

% set headers accordingly to columns in 'mass3' and write in sheet 1.
header = {'mono' 'av' 'scannr' 'nominal' 'nmd' 'nis' 'm/z' 'charge' 'Fr.Nr.'};
xlswrite(title, header, '1');

% write data starting at cell A2 on sheet 1.
xlswrite(title, mass3, '1', 'A2');
```

Published with MATLAB® R2014a
