# Supplementary material for: Screening Method for the Discovery of Potential Bioactive Cysteine-Containing Peptides Using 3D Mass Mapping
Source: J Am Soc Mass Spectrom. 2015 Nov 9;26(12):2039–50. doi: 10.1007/s13361-015-1282-z (PMC4654750; doi:10.1007/s13361-015-1282-z)
Supplement: Supplementary file 7 — (PDF 372 kb) [file 13361_2015_1282_MOESM7_ESM.pdf]

Script to generate 3D mass map 1
Erase all temporary files 1
Define FASTA file 1
Import FASTA file 2
Data generation 2
Generate the 3D mass map 3
3D mass map properties 4
Set view of 3D mass map 4

Script to generate 3D mass map

```
% The following script accompanies the article "Screening method for the
% discovery of potential bioactive cysteine-containing peptides using 3D
% mass mapping" and in particular Figure 1A, 1B, 1C and 1D.

% In this script, FASTA files containing protein/peptide sequences are
% imported into MATLAB. From these peptide sequences, the monoisiotopic,
% average and nominal masses are calculated using several MATLAB build-in
% functions from the Bioinformatics Toolbox.

% using the monoisotopic, nominal and average masses, the
% NMD and NIS of each sequence are calculated. Using the monoisotopic mass,
% NMD and NIS a 3D mass map is generated. Each sequence is colored
% according to the amount of cysteine residues present in its amino acid
% sequence.

% Disclaimer: the authors would like to state that this script has been
% used only with data obtained using an LTQ-Orbitrap Velos mass
% spectrometer (Thermo Scientific).

% For questions, clarification, tips or feedback try to seek
% contact through the MATLAB File Exchange
% (http://www.mathworks.com/matlabcentral/fileexchange/) or contact the
% authors of the corresponding article directly.
```

Erase all temporary files

```
% clear the workspace, close all windows and clear the command-window.

clear all
close all
clc
```

Define FASTA file

```
% Import the FASTA file. Be sure that all sequences in the FASTA file
% contain amino acid codes that are recognized by MATLAB to calculate the
% mass of the peptide. the following one-letter codes are NOT accepted:
% X, J, B, Z (because the mass is unspecified).
```

```
% Define the FASTA-file containing the peptide sequences:
file = 'your_FASTA_file.fasta';
```

Import FASTA file

```
% as a struct with two fields; the header and the corresponding sequence.
fastastruct = fastaread(file);

% The amino acid sequence is in the structure within apostrophs: ('SEQ').
% These apostrophs need to be removed, in order that the sequences is
% read SEQ instead of 'SEQ').


% loop over the length of the 'fastastruct'.
for i = 1:(length(fastastruct))

    % generate cell array 'Seq' that stores the sequence from the STRUCT
    % including the apostrophs
    Seq{i,1} = {fastastruct(i).Sequence};

    % generate the cell array 'seqs' that stores the sequences without the
    % apostrophs.
    seqs {i,1} = Seq{i,1}{:};

end % close the loop
```

Data generation

```
% For each peptide sequence, the monoisotopic mass, average mass and
% nominal mass can be calculated using MATLAB's 'isotopicdist' function.

% This function can generate high resolution isotope mass distribution (MD)
% and density function (DF), but these graphical representations will not
% be used here.

% The struct 'Info' generated for each sequence gives monoisotopic mass,
% average mass, most abundant mass, nominal mass, and the empirical
% formula for every input of string containing a valid amino acid sequence.

% loop over the length of 'seqs'
for i = 1:length(seqs)

    % Generate the struct containing mass parameters
    [~,Info] =  isotopicdist(seqs{i,1});

    % Create five new cell arrays to store information about masses, NIS and
    % NMD. For each sequence, store the sequence header in the first column of a
    % new cell array. In the second column, store the value corresponding to the
    % name of the cell array.

    % create array and store sequence header in the first column.
    Mono_mass {i,1} = {fastastruct(i).Header};
    Avg_mass {i,1} = {fastastruct(i).Header};
    Nominal_mass {i,1} = {fastastruct(i).Header};
    NIS {i,1} = {fastastruct(i).Header};
```

```
    NMD {i,1} = {fastastruct(i).Header};

    % Calculate and store the generated values. NIS and NMD are calculated
    % as explained in the corresponding article.

    % store the properties in the second column.
    Mono_mass {i,2} = Info.MonoisotopicMass(1,1);
    Avg_mass {i,2} = Info.CalculatedAverageMass(1,1);
    Nominal_mass {i,2} = Info.NominalMass(1,1);
    NIS {i,2} = 1000 * (Avg_mass{i,2}- Mono_mass{i,2} )/ Mono_mass{i,2};
    NMD {i,2} = 1000 * (Mono_mass{i,2} - Nominal_mass {i,2})/ Mono_mass{i,2};


end % close the loop
```

Generate the 3D mass map

```
% In the 3D mass map, sequences with 0, 1, 2, 3 and >4 cysteines are colored
% differently. The number of cysteine residues is calculated in this
% section.

% loop over the length of 'seqs'
for i = 1:length(seqs)

    % Count the amount of cysteine residues in a sequence.
    All = aacount(seqs{i,1});
    Cys = All.C;

        % If the sequence contains exactly 1 cysteine, color dark red
        % If the sequence contains exactly 2 cysteines, color red
        % If the sequence contains exactly 3 cysteines, color orange
        % If the sequence contains exactly 4 cysteines, color yellow
        % If the sequence contains  >4 cysteines, color white
        % else (e.g. if the sequence contains no cysteine) color black

        if  Cys < 2 && Cys > 0
                scatter3(NMD{i,2}, NIS {i,2}, Mono_mass{i,2}, 15, [0.5 0 0], 'filled'); hold
on;
            elseif Cys < 3 && Cys > 1
                scatter3(NMD{i,2}, NIS {i,2}, Mono_mass{i,2}, 15, [1 0 0], 'filled'); hold on;
            elseif Cys < 4 && Cys > 2
                 scatter3(NMD{i,2}, NIS {i,2}, Mono_mass{i,2}, 15, [1 0.5 0], 'filled'); hold
on;
            elseif Cys < 5 && Cys > 3
                 scatter3(NMD{i,2}, NIS {i,2}, Mono_mass{i,2}, 15, [1 1 0], 'filled'); hold
on;
            elseif Cys >4
                 scatter3(NMD{i,2}, NIS {i,2}, Mono_mass{i,2}, 15, [1 1 1], 'filled'); hold
on;
            else
                 scatter3(NMD{i,2}, NIS {i,2}, Mono_mass{i,2}, 15, 'k', 'filled'); hold on;
        end % close the loop

        % For reference, the color codes are given below.
        % These are RGB-values and can be changed accordingly.
```

```
        % 0 cys black [0 0 0]
        % 1 cys dark red [0.5 0 0]
        % 2 cys red [1 0 0]
        % 3 cys orange [1 0.5 0]
        % 4 cys yellow [1 1 0]
        % >4 cys white [1 1 1]

end % close the loop
```

3D mass map properties

```
% Get the handle to the figure and set the background color to white.
set(gcf, 'color', [1 1 1])

% Get the handle to the the current axis and set the color of the background
% to a shade of grey.
set(gca, 'color', [0.4 0.4 0.4])

% Get the handle to the the current axis and set the color of all three
% axis to black.
set(gca, 'XColor', 'k')
set(gca, 'YColor', 'k')
set(gca, 'ZColor', 'k')


% Label the x, y and z axis accordingly. The default fontsize of the labels
% is 10. set to size 12.
xlabel('NMD', 'FontSize', 12);
ylabel('NIS', 'FontSize', 12);
zlabel('mass (Da)', 'FontSize', 12);

% get the handle to the current axis and set fontsize to 12.
set(gca, 'FontSize', 12)

% set axis min and max values; in general NMD ranges from 0.3 to 0.7 and NIS
% ranges from 0.55 to 0.95. The mass of the peptides can vary, but in the
% file processed the largest peptide weighs 4274.9 Da.
axis([0.3 0.7 0.55 0.95 0 4500]);
```

Set view of 3D mass map

```
% default view is 'view(3)', as is used in Figure 1A in the corresponding
% article. Some 2D representational views are listed below.

% % For 2D view of NMD vs Mass, as is used in Figure 1B, use:
% view([0 -1 0])

% % For 2D view of NMD vs NIS, as is used in Figure 1C, use:
% view([0 0 1])

% % For 2D view of NIS vs Mass, as is used in Figure 1D, use:
% view([1 0 0])


% A legend can be added (as in Figure 1a). Due to MATLAB's plotting
```

```
% characteristics this is somewhat complicated. Use the following
% work-around:

% Make sure that in the original FASTA file, the first sequence
% contains 0 cysteines, the second sequence contains 1 cysteine, the third
% sequence contains 2 cysteines, the fourth sequence contains 3 cysteines,
% the fifth sequence containes 4 cysteines and the fifth sequence contains
% more than 4 cysteines.

% Add the legend
legend('0 cys','1 cys' ,'2 cys' ,'3 cys' ,'4 cys' ,'>4 cys' )
```

Published with MATLAB® R2014a
